# Upregulated UBE4B expression correlates with poor prognosis and tumor immune infiltration in hepatocellular carcinoma

**DOI:** 10.18632/aging.204414

**Published:** 2022-12-05

**Authors:** Xuyang Shao, Jun Zhu, Yanlong Shi, Hanlu Fang, Jingsi Chen, Yixiao Zhang, Jingyan Wang, Haokun Jian, Sheng Lan, Fei Jiang, Fei Zhong, Yewei Zhang, Chenxi Cao

**Affiliations:** 1 Graduate School of Bengbu Medical College, Bengbu 233000, Anhui Province, China; 2 Department of General Surgery, The Second Affiliated Hospital of Jiaxing University, Jiaxing 314000, Zhejiang Province, China; 3 Department of Oncology, Fuyang Hospital of Anhui Medical University, Fuyang 236000, Anhui Province, China; 4 Hepatopancreatobiliary Center, The Second Affiliated Hospital of Nanjing Medical University, Nanjing 210003, Jiangsu Province, China; 5 Institute of Medical and Health Science of HeBMU, Hebei Medical University, Shijiazhuang 050017, Hebei Province, China; 6 Department of Intensive Care Unit, Fuyang Women and Children’s Hospital, Fuyang 236000, Anhui Province, China; 7 The First Clinical College of Zhejiang Chinese Medical University, Hangzhou 310053, Zhejiang Province, China; 8 Department of Anesthesia, Shaoxing People’s Hospital, Shaoxing 312000, Zhejiang Province, China; 9 School of Basic Medical Sciences, Xinxiang Medical University, Xinxiang 453003, Henan Province, China; 10 The Second Clinical College of Guangzhou Medical University, Guangzhou 510030, Guangdong Province, China; 11 Department of General Surgery, Fuyang Hospital of Anhui Medical University, Fuyang 236000, Anhui Province, China; 12 Department of Oncology, The First Affiliated Hospital of Anhui Medical University, Hefei 230000, Anhui Province, China

**Keywords:** hepatocellular carcinoma, ubiquitin ligase, prognosis-related biomarker, pan-cancer analysis, competing endogenous RNA

## Abstract

Background: Hepatocellular carcinoma (HCC) is a major human health concern. Increasing evidence has demonstrated that ubiquitin ligase E4B (UBE4B) may be involved in the occurrence and development of various human cancers and may affect prognosis. However, the specific role and mechanism of UBE4B in HCC is unclear.

Methods: A pan-cancer analysis of UBE4B expression, clinicopathological features, and prognosis was performed using bioinformatics techniques. Subsequently, the expression, prognosis, and correlation of UBE4B and its upstream miRNAs and lncRNAs were analyzed. We investigated the relationship between UBE4B expression and immune cell infiltration, immunomodulatory factors, and chemokines in HCC. The expression levels of UBE4B and its upstream lncRNAs (FGD5-AS1, LINC00858, and SNHG16) and miRNAs (hsa-miR-22-3p) were evaluated in HCC cell lines using qRT-PCR.

Results: UBE4B expression increased in HCC and was correlated with a poor survival rate in patients with HCC. A ceRNA network was established to identify the UBE4B-hsa-miR-22-3p-FGD5-AS1/LINC00858/SNHG16 regulatory axis in HCC. UBE4B expression was significantly associated with immune cell infiltration, immunomodulators, chemokines, and their receptors in HCC. The mRNA expression of FGD5-AS1, LINC00858, SNHG16, and UBE4B was higher in the HCC cell lines (7721 and HepG2) than in the normal hepatocyte line (LO2), and the expression of hsa-miR-22-3p mRNA showed a decreasing trend.

Conclusions: Our findings showed that upregulation of UBE4B was associated with poor prognosis and tumor immune infiltration in HCC. These findings will aid in understanding the relevant functions of UBE4B and provide new strategies for drug development and exploration of prognosis-related biomarkers.

## INTRODUCTION

Hepatocellular carcinoma (HCC) is the most common pathological type of liver cancer and the leading cause of death in patients with liver cancer [1, 2]. The incidence and mortality rates of liver cancer worldwide rank 6^th^ and 3^rd^ of all cancers, respectively, with approximately 960,000 new cases and 830,000 deaths per year [3]. The main risk factors of HCC are hepatitis B virus (HBV) infection, hepatitis C virus (HCV) infection, alcohol consumption, obesity, diabetes, non-alcoholic fatty liver disease, aflatoxins, etc. [4, 5]. Although significant progress has been made in treating HCC with surgery, chemotherapy, targeted therapy, immunotherapy, and interventional therapy, the early symptoms of HCC are atypical, and most patients are at advanced stages when diagnosed. At this time, the available treatments are limited, resulting in a poor prognosis with a 5-year survival rate of approximately 18.4% [6, 7]. Therefore, it is importance to explore more effective therapeutic targets and identify novel prognostic markers to improve HCC survival.

Protein post-translational modifications (PTMs) are processes that employ reversible state patterns to modulate protein function and alter physicochemical properties [8]. Studies have identified more than 400 post-translational modifications, with common modifications including ubiquitination, sumoylation, phosphorylation, acetylation, and methylation [9]. The ubiquitin-proteasome system (UPS) is a common post-translational modification pathway that is involved in the regulation of cell survival and differentiation [10]. Ubiquitination requires the participation of three enzymes, namely ubiquitin-activating enzyme (E1), ubiquitin-binding enzyme (E2), and ubiquitin-protein ligase (E3) [11]. UBE4B belongs to the U-box family of ubiquitin ligases, also known as UFD2a, which is a novel E3 [12]. Many studies have investigated the substrate function of UBE4B, and it has been confirmed that it is involved in the ubiquitinated degradation of important proteins such as EGFR, p53, and caspase3 [13, 14]. Additionally, UBE4B can be involved in the development of various cancers, such as renal cell carcinoma, breast cancer, and neural tube cell tumors, by regulating the ubiquitination of substrate proteins [15–17]. These results suggest that UBE4B may be a novel prognostic indicator in patients with multiple malignancies.

In this study, we investigated the UBE4B expression, prognosis, tumor immune microenvironment, and regulatory mechanisms of HCC. Next, we explored non-coding RNA regulation associated with UBE4B and constructed a critical UBE4B-miRNA-LncRNA regulatory axis. These findings will aid us to understand the relevant functions of UBE4B and provide new strategies for drug development and exploration of prognosis-related biomarkers.

## MATERIALS AND METHODS

### Data acquisition

The expression of UBE4B was evaluated in 33 cancer and normal tissues using the The Cancer Genome Atlas (TCGA) (https://www.cancer.gov/tcga) and The Genotype-Tissue Expression (GTEx) (http://commonfund.nih.gov/GTEx/) databases. RNA sequencing data and clinical follow-up information of 33 patients with cancer were obtained from the TCGA cohort. All expression data were normalized using a log2 transformation.

### TIMER database analysis

Tumor Immune Estimation Resource (TIMER) (https://cistrome.shinyapps.io/timer/) is a web server for analyzing the abundance of tumor infiltrates [18]. It was used to investigate the expression and abundance of immune infiltration in different cancers based on the TCGA cohort, with the threshold value as log2TPM.

### GEPIA database analysis

Gene Expression Profiling Interactive Analysis (GEPIA) (http://gepia.cancer-pku.cn/) is a web tool for cancer and normal gene-expression profiling and interactive analysis using TCGA and GTEx cohorts [19]. Analysis of violin images of UBE4B in various pathological stages were performed using the Pathological Stage Map module in the GEPIA database.

### Kaplan–Meier plotter analysis

The relationship between UBE4B expression and prognosis, including overall survival (OS) and recurrence-free survival (RFS), was examined using the Kaplan–Meier plotter (http://kmplot.com/analysis/) in different cancer types. Hazard ratios (HRs) and 95% confidence intervals (CIs) were calculated along with the log-rank *p*-value.

### Competing endogenous RNAs (CeRNA) network construction

TarBase V.8 (https://dianalab.e-ce.uth.gr/html/diana/web/index.php?r=tarbasev8) was used to predict the miRNA targets that bind to UBE4B. The lncRNAs upstream of these miRNAs were identified using miRNet (https://www.mirnet.ca/miRNet/home.xhtml). StarBase (https://starbase.sysu.edu.cn/index.php) was used to predict miRNA and lncRNA expression levels and their prognostic values. Additionally, starBase was used to perform expression correlation analysis of hsa-miR-22-3p-UBE4B, lncRNA-hsa-miR-22-3p, and lncRNA-UBE4B in HCC.

### TISIDB database analysis

TISIDB (http://cis.hku.hk/TISIDB/index.php) is an online platform for the interaction between tumors and the immune system that integrates multiple heterogeneous data types [20]. The TISIDB database was used investigated the relationship between UBE4B and 45 immunostimulants, 24 immunosuppressive agents, 41 chemokines, and 18 receptors in HCC.

### Cell culture

The human liver (LO2) and human HCC (7721 and HepG2) cell lines were purchased from Cell Bank (Shanghai, China). All cell lines were cultured in Dulbecco’s modified Eagle’s medium (DMEM; cat no.SH30022.01; HyClone) supplemented with 10% fetal bovine serum (FBS; cat no.2127186; VivaCell) and 1% penicillin and streptomycin (cat no.15140122; Gibco) at 37° C in 5% CO_2_.

### Quantitative real-time PCR (qRT-PCR)

Total RNA was extracted from the human liver (LO2) and human HCC (7721 and HepG2) cell lines using TRIzol reagent (cat no.12183-555; Invitrogen). Reverse transcription of cDNA to cDNA using PrimeScript™ RT Master Mix kit (cat no.RR036A; Takara). qRT-PCR analysis was performed using TB Green® Premix Ex Taq™ II (cat no.RR820A; Takara). The primers used were as follows: UBE4B forward:5′-CTACCTCCCCAATAGGTGCAT-3′ and UBE4B reverse:5′-GGCGAGCTGCTGAGAGAAC-3′; FDG5-AS1 forward:5′-GAAGGGCCGAAGAGCTCAAT-3′ and FDG5-AS1 reverse:5′-GGCTCGCAAAGTGTCTGTTG-3′; LINC00858 forward:5′-CCCAGCTCCTTACACACGTT-3′ and LINC00858 reverse:5′-TTCAGAGGCCTGCATCACTG-3′; SNHG16 forward:5′-CAGAATGCCATGGTTTCCCC-3′ and SNHG16 reverse:5′-TGGCAAGAGACTTCCTGAGG-3′; Has-miR-22-3P forward:5′-TCAGTGCATCACAGAACTTTGT-3′ and Has-miR-22-3P reverse:5′-TGGCAAGAGACTTCCTGAGG-3′; Actin forward:5′-GTGGCCGAGGACTTTGATTG-3′ and Actin reverse:5′-CCTGTAACAACGCATCTCATATT-3′. Relative quantification was performed using the 2^−ΔΔCt^ method.

### HPA database analysis

The Human Protein Atlas (HPA) (www.proteinatlas.org/) was used to detect the protein expression of UBE4B in hepatocellular carcinoma and corresponding normal tissues in the “tissue” and “pathology” modules. All images were confirmed by immunohistochemical experiments and specific patient information is listed.

### Statistical analysis

All data analyses were automatically performed using the online database mentioned above. Log-rank *p*-value <0.05 or *p*-value <0.05 were considered statistically significant.

### Availability of data and materials

All data generated or analyzed during this study are included in this article.

## RESULTS

### Identification of UBE4B expression in pan-cancer

**Figure 1:**
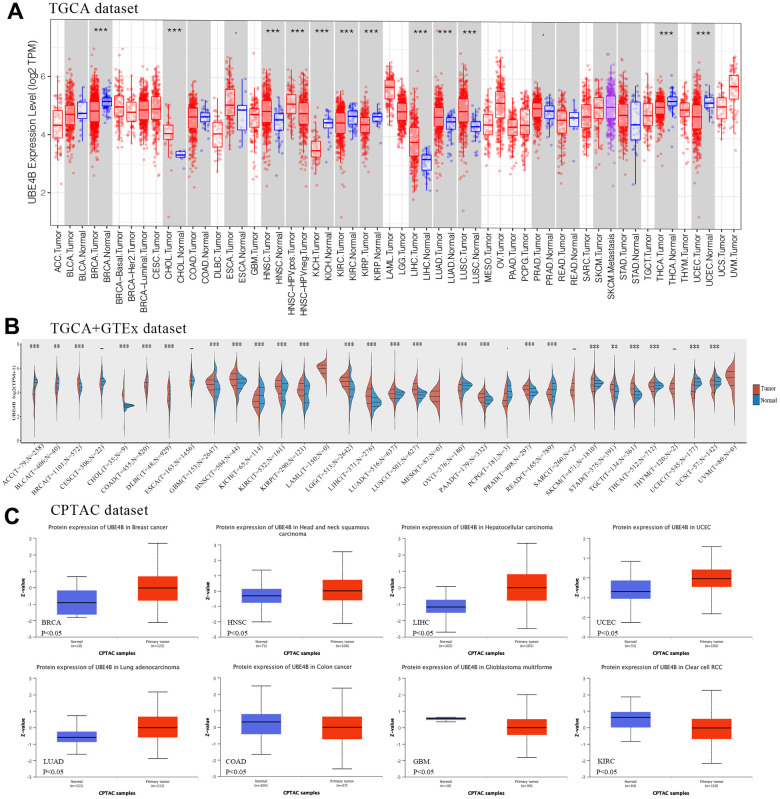
**Expression of UBE4B in pan-cancer.** (**A**) UBE4B expression in normal and cancer tissues in TIMER. (**B**) UBE4B expression in TCGA cancers compared with corresponding TCGA and GTEx normal tissues. (**C**) The UBE4B protein expression was determined by the CPTAC dataset in BRCA, HNSC, LIHC, UCEC, LUAD, COAD, GBM, and KIRC. *P < 0.05; **P < 0.01; ***P < 0.001.

The expression levels of UBE4B in tumors and normal tissues were investigated. The results suggested that UBE4B mRNA was overexpressed in Cholangiocarcinoma (CHOL), Head and Neck Squamous Cell Carcinoma (HNSC), Liver Hepatocellular Carcinoma (LIHC), Lung Adenocarcinoma (LUAD), and Lung Squamous Cell Carcinoma (LUSC) using the TIMER database and downregulated in Breast Invasive Carcinoma (BRCA), Kidney Chromophobe (KICH), Kidney Renal Clear Cell Carcinoma (KIRC), Kidney Renal Papillary Cell Carcinoma (KIRP), Thyroid Carcinoma (THCA), and Uterine Corpus Endometrial Carcinoma (UCEC) (Figure 1A). Then, samples were integrated from the GTEx database and the expression levels of UBE4B in various cancers were compared. UBE4B mRNA was upregulated in BRCA, CHOL, Lymphoid Neoplasm Diffuse Large B-cell Lymphoma (DLBC), HNSC, KIRC, KIRP, LIHC, LUAD, LUSC, Ovarian Serous Cystadenocarcinoma (OV), Pancreatic Adenocarcinoma (PAAD), Prostate Adenocarcinoma (PRAD), Skin Cutaneous Melanoma (SKCM), Stomach Adenocarcinoma (STAD), and Testicular Germ Cell Tumors (TGCT) and downregulated in Adrenocortical Carcinoma (ACC), Bladder Urothelial Carcinoma (BLCA), Colon Adenocarcinoma (COAD), Glioblastoma Multiforme (GBM), KICH, Brain Lower Grade Glioma (LGG), Rectum Adenocarcinoma (READ), THCA, and Uterine Carcinosarcoma (UCS) (Figure 1B). In the CPTAC database, UBE4B protein expression was markedly increased in BRCA, HNSC, LIHC, UCEC, and LUAD, and decreased in COAD, GBM, and KIRC compared to normal tissues (Figure 1C).

### Clinicopathology and prognostic value of UBE4B in pan-cancer

**Figure 2:**
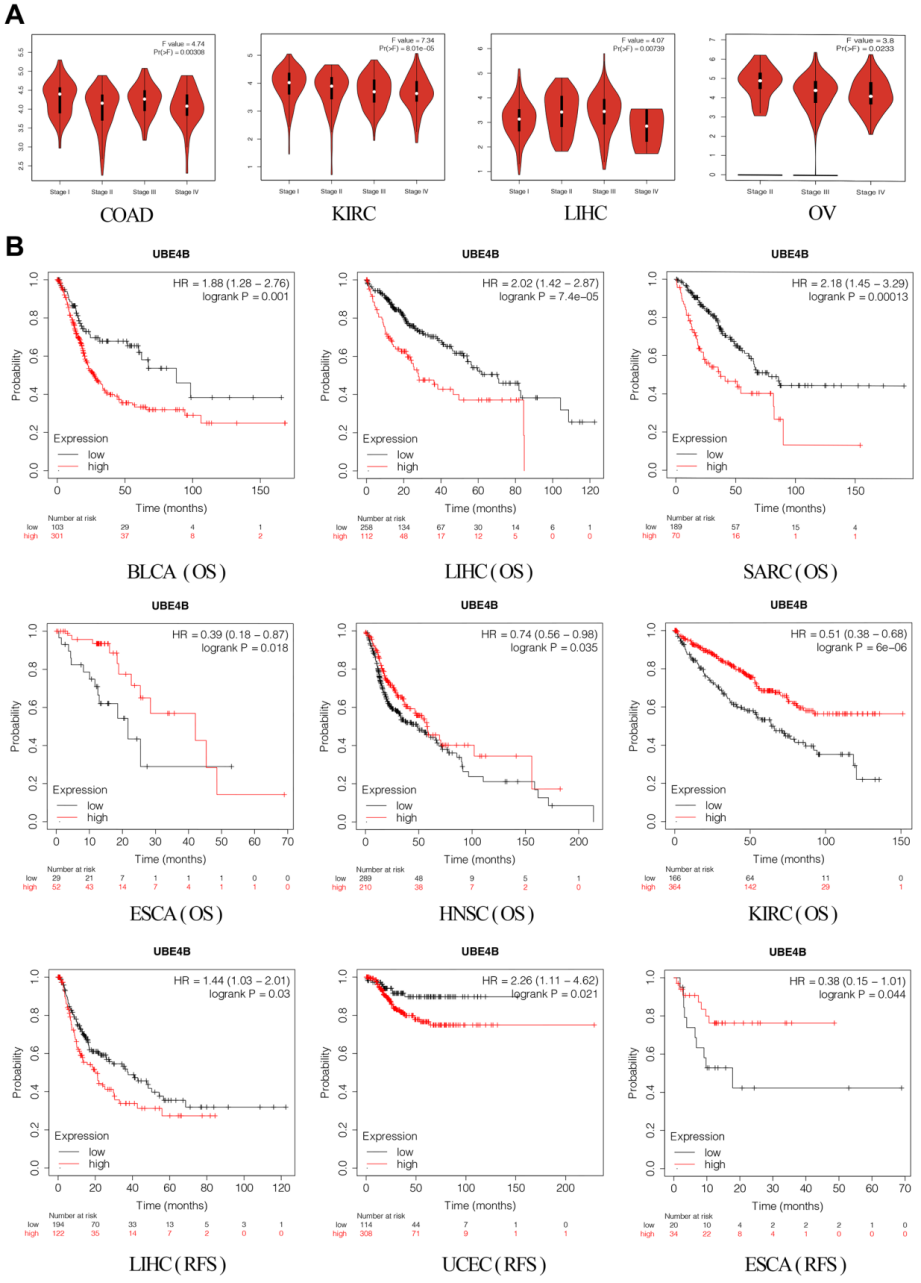
**Correlation of UBE4B expression with pathological staging and prognosis.** (**A**) Expression of UBE4B in pathological stages (stage I, stage II, stage III, and stage IV) of COAD, KIRC LIHC, and OV. (**B**) The OS and RFS plot of UBE4B in different cancers by Kaplan-Meier Plotter.

The GEPIA database was used to elucidate the relationship between UBE4B expression and pathological stages in different cancers, revealing significant correlations between UBE4B expression and pathological stages of COAD (*P* = 0.00308), KIRC (*P* = 8.01 × 10^-5^), LIHC (*P* = 0.00739), and OV (*P* = 0.0233) (Figure 2A). Next, pan-cancer survival analysis for UBE4B was performed. The OS results indicated that patients with BLCA (*P* = 0.001), LIHC (*P* = 7.4 × 10^-5^), and Sarcoma (SARC) (*P* = 0.00013) with high UBE4B expression exhibited a worse prognosis, while patients with Esophageal Carcinoma (ESCA) (*P* = 0.018), HNSC (*P* = 0.035), and KIRC (*P* = 6 × 10^-6^) exhibited an improved prognosis. Moreover, UBE4B overexpression was correlated with inferior prognosis in patients with LIHC (*P* = 0.03) and UCEC (*P* = 0.021) but with superior prognosis in patients with ESCA (*P* = 0.044) (Figure 2B).

### Forecast and evaluation of the upstream miRNAs of UBE4B

**Figure 3:**
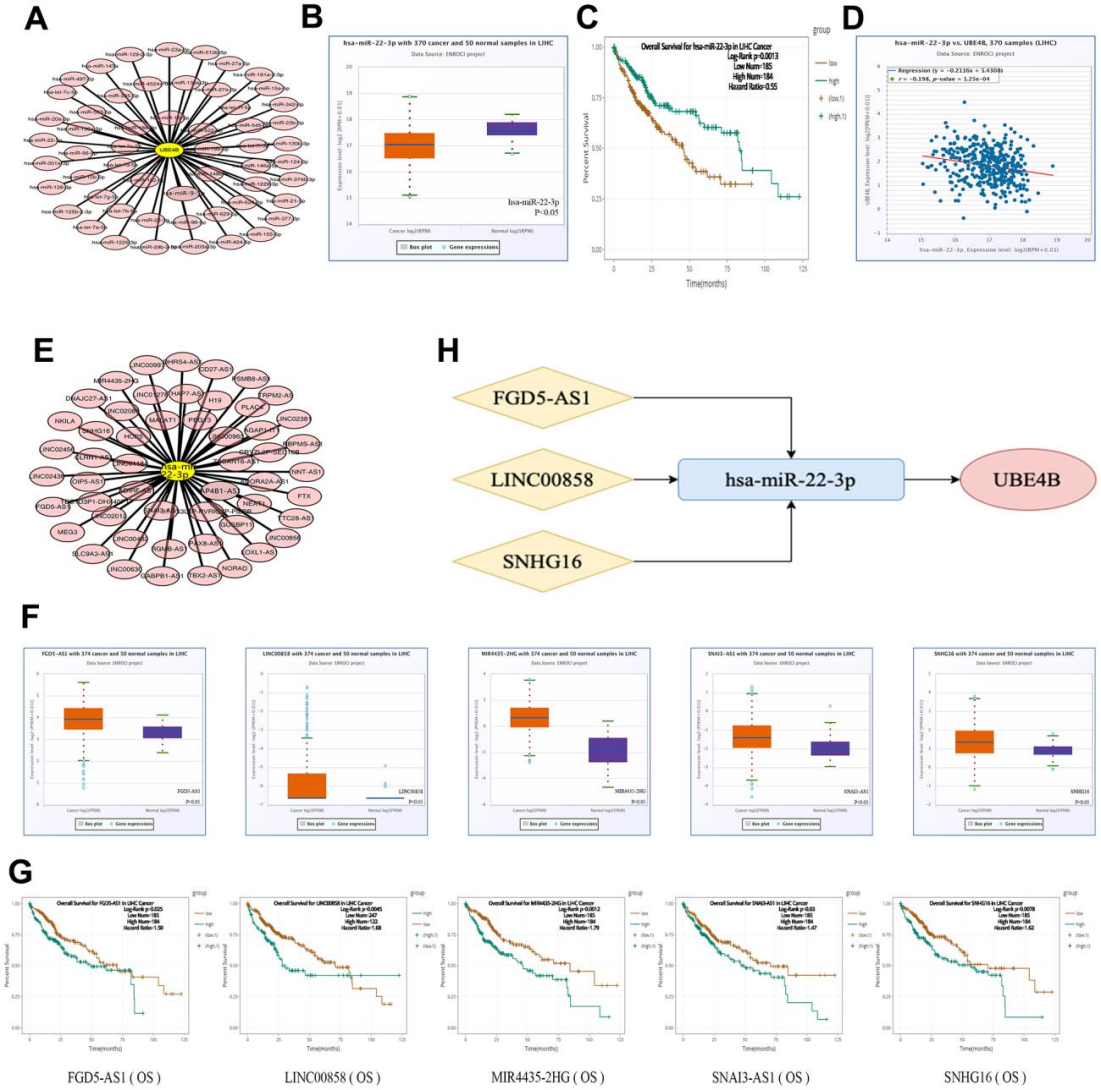
**Construction of ceRNA network.** (**A**) The miRNA-UBE4B regulatory network was developed by cytoscape software. (**B**–**D**) The expression (**B**), OS (**C**) and correlation with UBE4B (**D**) of hsa-miR-22-3p in HCC by StarBase database. (**E**) The lncRNA-hsa-miR-22-3p regulatory network constructed by cytoscape software. (**F**, **G**) The expression (**F**) and OS (**G**) of FGD5-AS1, LINC00858, MIR4435-2HG, SNAI3-AS1 and SNHG16 by StarBase database. (**H**) The schematic diagram of potential ceRNA network regulation axis.

The miRNAs upstream of UBE4B was predicted using the TarBase V.8 database, and 55 miRNAs were retrieved (Figure 3A). In accordance with the ceRNA hypothesis, we estimated the predicted miRNA expression patterns and prognostic value in LIHC using the starBase database. The results indicated that only downregulation of hsa-miR-22-3p was correlated with worse OS in patients with LIHC (*P* = 5.4 × 10^-10^; *P* = 0.0013) (Figure 3B, 3C). A correlation analysis of UBE4B and hsa-miR-22-3p was conducted (Figure 3D), indicating that the hsa-miR-22-3p/UBE4B axis was involved in the ceRNA mechanism. Therefore, hsa-miR-22-3p could be considered as a key miRNA for follow-up exploration.

### Prediction and validation of upstream lncRNAs of hsa-miR-22-3p

The online miRNet database was used to predict the upstream lncRNA of hsa-miR-22-3p. A total of 52 lncRNAs were identified (Figure 3E). A ceRNA-based hypothesis argues that lncRNAs negatively regulate miRNAs, while positively modulating mRNA [21]. Moreover, we evaluated the expression and prognosis of the 52 lncRNAs using the starBase database. The expression levels of five lncRNAs (MIR4435-2HG, FGD5-AS1, LINC00858, SNAI3-AS1, and SNHG16) was significantly upregulated and associated with adverse prognosis in patients with LIHC (*P* < 0.05) (Figure 3F, 3G). Additionally, we analyzed the correlation between five lncRNAs and hsa-miR-22-3p or UBE4B using the starBase database and revealed that three lncRNAs (FGD5-AS1, LINC00858, and SNHG16) were inversely co-expressed with hsa-miR-22-3p and positively co-expressed with UBE4B (Table 1). We constructed a potential FGD5-AS1/LINC00858/SNHG16/hsa-miR-22-3p/UBE4B regulation axis, which may play a vital role in the development and progression of LIHC (Figure 3H).

**Table 1:** Correlation analysis between lncRNA and hsa-mir-22-3p or lncRNA and UBE4B in HCC determined by starBase database.

**lncRNA**	**miRNA**	***R* value**	***p* value**
MIR4435-2HG	hsa-mir-22-3p	-0.168	1.16E-03**^a^
FGD5-AS1	hsa-mir-22-3p	-0.304	2.30E-09***^a^
LINC00858	hsa-mir-22-3p	-0.196	1.47E-04***^a^
SNAI3-AS1	hsa-mir-22-3p	-0.169	1.07E-03**^a^
SNHG16	hsa-mir-22-3p	-0.382	2.62E-14***^a^
**lncRNA**	**mRNA**	***R* value**	***p* value**
MIR4435-2HG	UBE4B	-0.046	3.74E-01
FGD5-AS1	UBE4B	0.53	1.61E-28***^a^
LINC00858	UBE4B	0.275	6.53E-08***^a^
SNAI3-AS1	UBE4B	-0.003	9.47E-01
SNHG16	UBE4B	0.266	1.70E-07***^a^

^a^These results are statistically significant.

**P value < 0.01;***P value <0.001.

### Correlation between immune infiltration and UBE4B expression in LIHC

**Figure 4:**
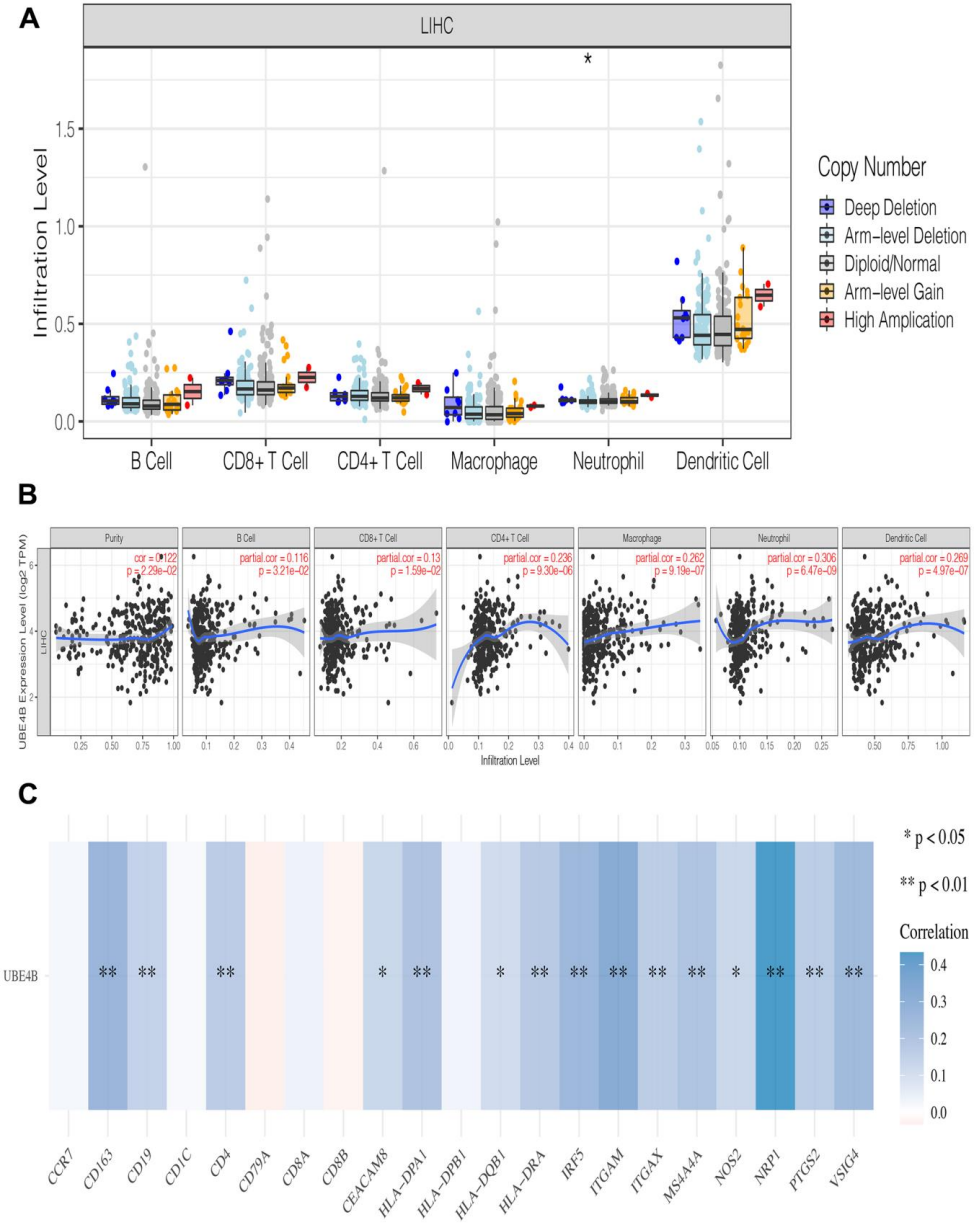
**The relationship of immune cell infiltration with UBE4B level in HCC.** (**A**) The infiltration level of various immune cells in HCC with different copy numbers of UBE4B. (**B**) The correlation of UBE4B expression level with B cell, CD8+ T cell, CD4+ T cell, macrophage, neutrophil, or dendritic cell infiltration level in HCC. (**C**) Correlation analysis between UBE4B and biomarkers of immune cells in HCC. *P < 0.05; **P < 0.01.

Immune infiltration plays a pivotal role in tumor pathogenesis [22]. Immune cell infiltration did not change significantly at different UBE4B copy numbers in LIHC (Figure 4A). The association between UBE4B expression and the degree of immune cell infiltration was evaluated in LIHC using the TIMER database. A significant positive correlation was observed between UBE4B expression and immune cells in LIHC, including B cells (*P* = 3.21 × 10^-2^), CD8+ T cells (*P* = 1.59 × 10^-2^), CD4+ T cells (*P* = 9.30 × 10^-6^), macrophages (*P* = 9.19 × 10^-7^), neutrophils (*P* = 6.47 × 10^-9^), and dendritic cells (*P* = 4.97 × 10^-7^) (Figure 4B). Furthermore, the correlation between UBE4B expression and immune cell biomarkers was evaluated in patients with LIHC. A significant positive correlation was observed between UBE4B and most immune cell biomarkers in LIHC, in addition to CCR7, CD1C, CD79A, CD8A, CD8B, and HLA-DPB1 (Figure 4C).

### Relationship between UBE4B expression and immune modulators in LIHC

**Figure 5:**
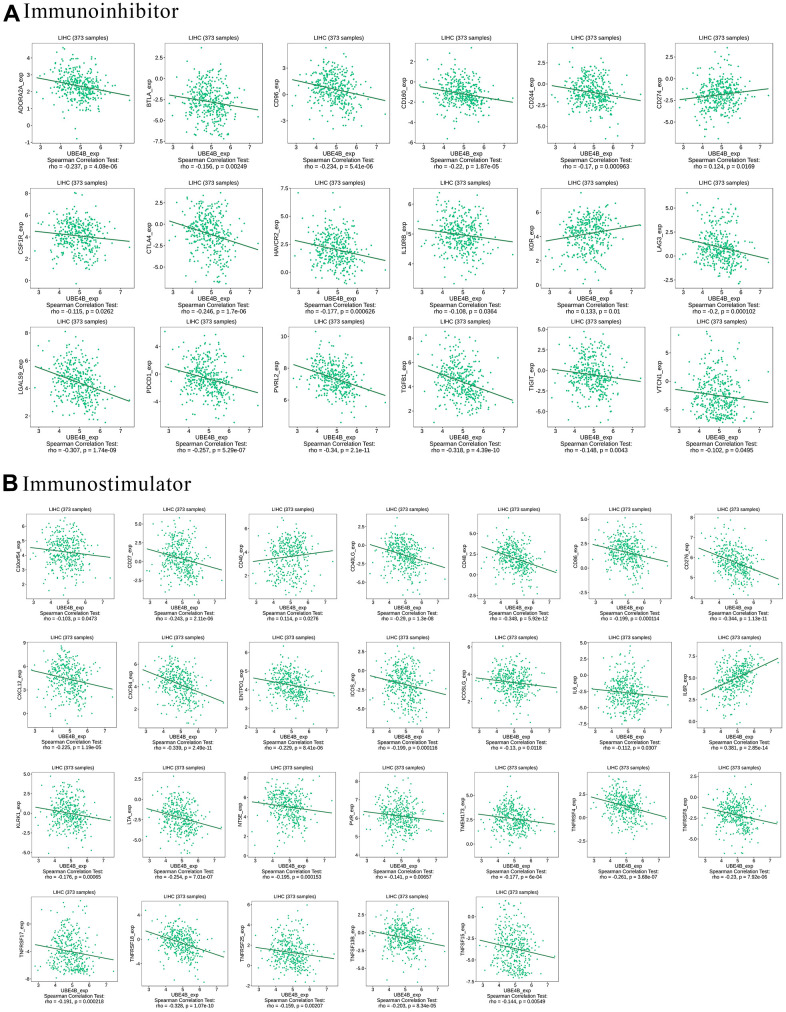
**Correlation between UBE4B expression and immunomodulators in HCC by TISIDB database.** (**A**) Immunoinhibitors, (**B**) immunostimulators.

Immune modulators affect the function of the immune system [23]. UBE4B was significantly associated with most immune inhibitors, including ADORA2A, BTLA, CD96, CD160, CD244, CD274, CSF1R, CTLA4, HAVCR2, IL10RB, KDR, LAG3, LGALS9, PDCD1, PVRL2, TGFB1, TIGIT, and VTCN1 (*P* < 0.05) (Figure 5A). Moreover, UBE4B expression was closely associated with the following immune stimulators: C10orf54, CD27, CD40, CD40LG, CD48, CD86, CD276, CXCL12, CXCR4, ENTPD1, ICOS, ICOSLG, IL6, IL6R, KLRK1, LTA, NT5E, PVR, TMEM17, TNFRSF4, TNFRSF8, TNFRSF17, TNFRSF18, TNFRSF25, TNFSF13B, and TNFSF15 (*P* < 0.05) (Figure 5B). These results indicate the possible involvement of UBE4B in modulating tumor immune escape.

### Relationship between UBE4B expression and chemokines in LIHC

**Figure 6:**
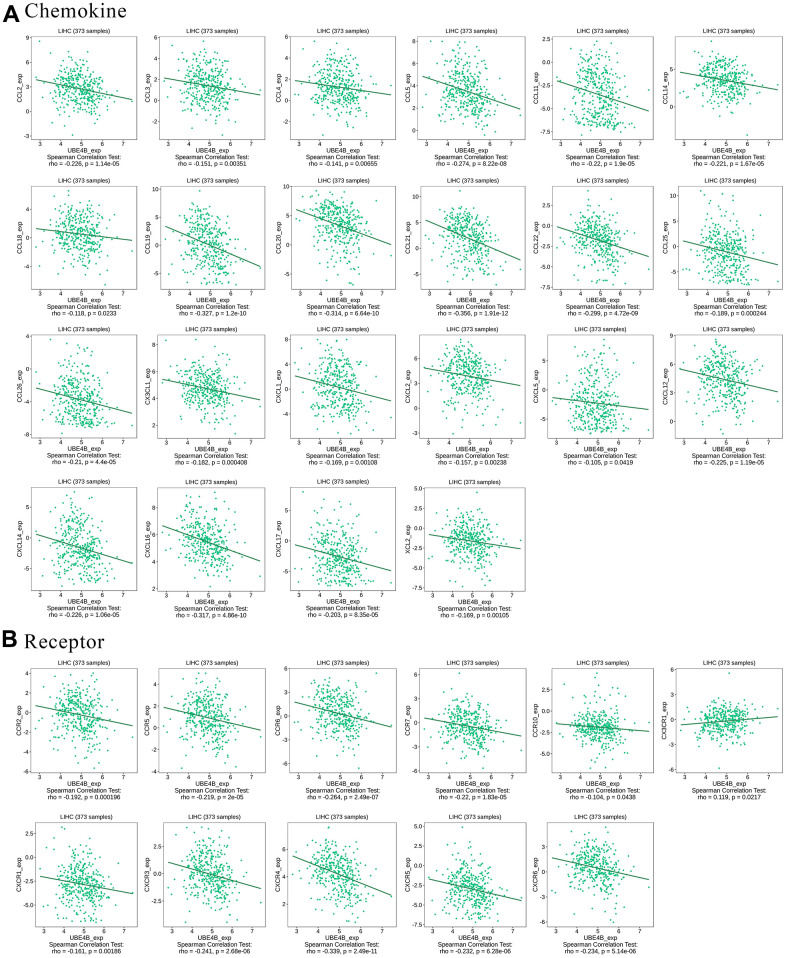
**Correlation between UBE4B expression and chemokines in HCC by TISIDB database.** (**A**) Chemokines, (**B**) chemokine receptors.

Chemokines enable directed chemotaxis of immune cells [24]. UBE4B expression was significantly associated with chemokines such as CCL2, CCL3, CCL4, CCL5, CCL11, CCL14, CCI18, CCL19, CCL20, CCL21, CCL22, CCL25, CCL26, CX3CL1, CXCL1, CXCL2, CXCL5, CXCL12, CXCL14, CXCL16, CXCL17, and XCL2 (*P* < 0.05) (Figure 6A). We further investigated the correlation between UBE4B and chemokine receptor expression. We found that UBE4B expression was significantly linked to CCR2, CCR5, CCR6, CCR7, CCR10, CX3CR1, CXCR1, CXCR3, CXCR4, CXCR5, and CXCR6 expression (*P* < 0.05) (Figure 6B). These results further demonstrated that UBE4B is involved in immune modulation in LIHC.

### Verification of the mRNA and protein expression of genes

**Figure 7:**
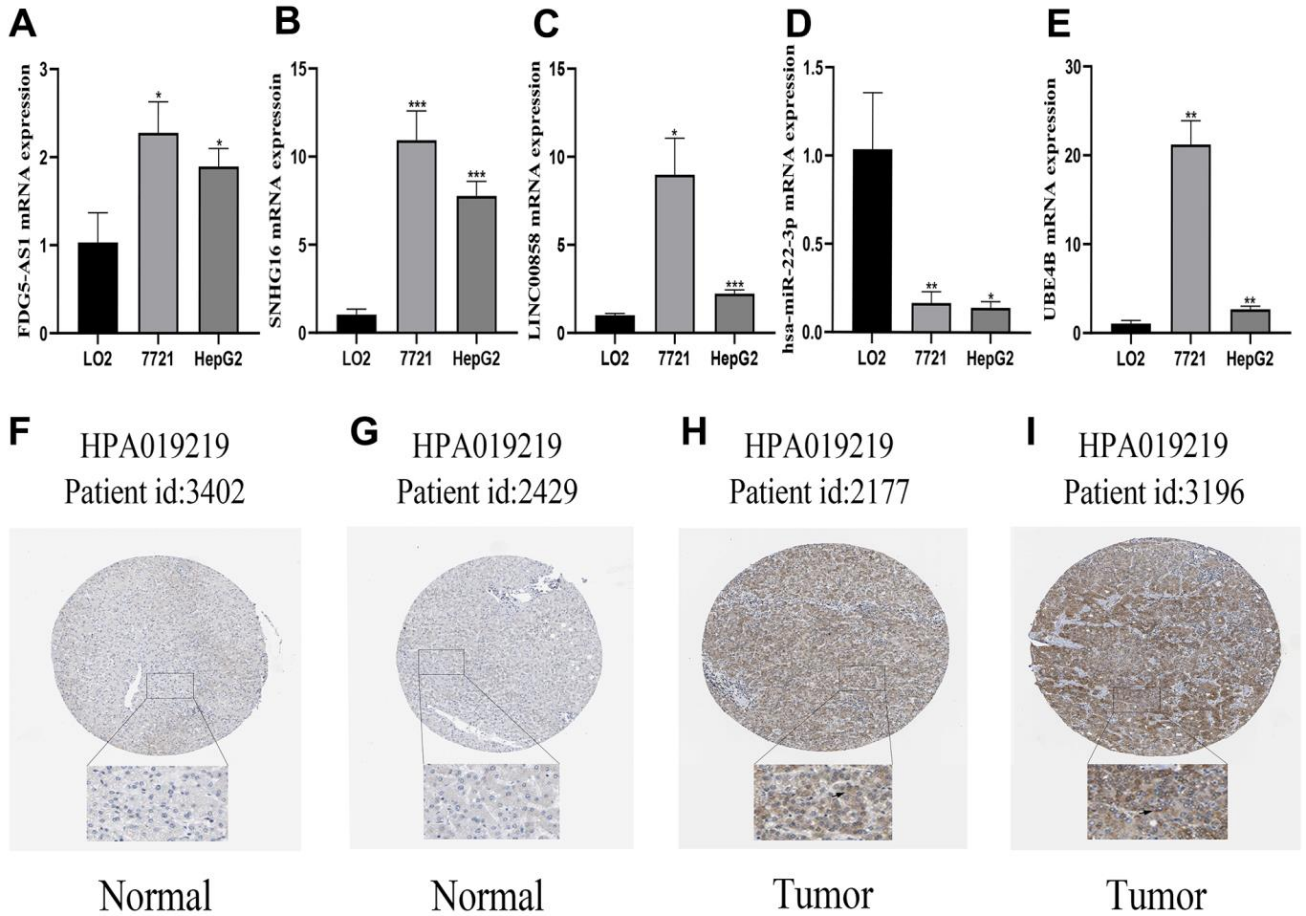
**Verification of the mRNA and protein expression of genes.** (**A**–**E**) qRT-PCR analysis detected the expression of FGD5-AS1, SNHG16, LINC00858, hsa-miR-22-3p, and UBE4B in normal cell line (LO2), and HCC cell lines (7721, HepG2). (**F**–**I**) Verification of the protein expression of UBE4B in HCC and corresponding normal tissues by HPA database. *P < 0.05; **P < 0.01; ***P < 0.001.

The mRNA expression levels of FGD5-AS1, LINC00858, SNHG16, hsa-miR-22-3p, and UBE4B were evaluated in HCC cell lines. The mRNA expression levels of FGD5-AS1, LINC00858, SNHG16, and UBE4B were elevated in the HCC cell lines (7721 and HepG2) compared to the normal liver cell line (LO2), while the mRNA expression of hsa-miR-22-3p showed a decreasing trend (Figure 7A–7E). Furthermore, we explored the protein expression of UBE4B in HCC using the HPA database. The results revealed that UBE4B protein expression was higher in HCC tissues than in corresponding normal tissues, mainly in the cytoplasm and cellular membranes (Figure 7F–7I).

## DISCUSSION

HCC has been a problem affecting human health for many years. There are observable variations in tumor proliferation, invasion, and metastasis between different HCC subtypes; therefore, it is necessary to further investigate biological molecular regulation in HCC. UBE4B is a U-box family of E4-active ubiquitin ligases that determine the specific recognition of substrates [16]. UBE4B can act as an oncogene or tumor suppressor gene in different types of cancers with opposite functions [13, 25]. Zhang et al. showed that UBE4B influenced the development of HCC by promoting the growth of HCC cells, and silencing of UBE4B inhibited the proliferation, migration, and invasion of HCC cells, resulting in significant apoptosis [26]. However, a regulatory axis related to UBE4B in HCC is still lacking and requires detailed exploration.

In this study, the expression of UBE4B was analyzed in pan-cancer using TCGA and GTEx databases. UBE4B expression was found to be increased at both mRNA and protein levels in HCC compared with normal samples, and was associated with clinicopathological characteristics in the survival analysis. OS analysis also indicated the prognostic value of UBE4B in several cancers, including BLCA, LIHC, SARC, ESCA, HNSC, and KIRC. A previous study demonstrated that UBE4B expression is often upregulated at the transcriptional (71%) and translational (84%) levels in HCC tissues [16]. This report was consistent with our results, which illustrate the carcinogenic effects of UBE4B in HCC.

The correlation between UBE4B expression and HCC progression was investigated. UBE4B upstream miRNA expression patterns, more specifically hsa-miR-22-3p, also affected HCC in the starBase database. Recent studies have found that hsa-miR-22-3p acts as an important regulatory gene in breast cancer [27], Japanese encephalitis [28], and obesity [29]. The expression and prognosis of the upstream lncRNA of hsa-miR-22-3p, as a negative precursor molecule for miRNA, was analyzed using the starBase database, and five related lncRNAs (MIR4435-2HG, FGD5-AS1, LINC00858, SNAI3-AS1, and SNHG16) were shown to be significantly upregulated in HCC and associated with poor prognosis in patients with HCC. This is a top-down, molecular regulatory mechanism.

P53 protein, a well-known substrate of UBE4B, has been shown to regulate cell cycle arrest, apoptosis, and DNA repair processes, which play critical roles in preventing tumor progression [30, 31]. This indicates that the overexpression of UBE4B leads to a carcinogenic process, with the amount of p53 protein greatly reduced in hepatocytes. Moreover, HCC cells have evolved to transfer oncogenic ncRNAs to recipient cells via exosomes, steadily transmitting biological signals and promoting tumor growth [32, 33]. Similarly, it has been reported that miRNAs may also regulate the activity and function of p53 by directly targeting UBE4B [34]. However, there is no clear evidence for a relationship between hsa-miR-22-3p and tumorigenesis. Our results demonstrated a tumor process between UBE4B and HCC. Therefore, we propose a novel regulatory axis for targeted drugs, namely UBE4B-hsa-miR-22-3p-FGD5-AS1/LINC00858/SNHG16. Moreover, we confirmed that the mRNA expression of FGD5-AS1, LINC00858, SNHG16, and UBE4B was higher in HCC cell lines than that in normal hepatocyte lines, and the expression of hsa-miR-22-3p mRNA showed a decreasing trend.

Non-coding RNAs are important components of exosomes, and can regulate the TME and promote HCC progression, including development, progression, infiltration, metastasis, and angiogenesis [35]. These are traits of tumor immuno-infiltration and immune escape. Moreover, oncogenic ncRNAs from exosomes may also suppress the activity of immunosuppressive cells such as T-effector cells, ultimately leading to local or systemic immunosuppression. Our study showed the same mechanism, as evidenced by all the immune cells analyzed in HCC, including B cells, CD8+ T cells, CD4+ T cells, macrophages, neutrophils, and dendritic cells. Furthermore, UBE4B expression was significantly and positively correlated with most immune cell biomarkers in HCC.

The degree of the immune response is regulated by the balance between the immunoinhibitor and immunostimulator pathways. These receptors and ligands are known as immune checkpoints [36]. Our study showed that UBE4B is significantly associated with immunoinhibitors and immunostimulators in HCC. The expression of UBE4B was significantly associated with many chemokines and chemokine receptors, suggesting that UBE4B may be associated with immune regulation in HCC and that these molecules could be potential immunotherapeutic targets for UBE4B in HCC.

The current study had some limitations. First, most data are based on online databases that are constantly updated and expanded, which may affect the results of the study. Second, this study requires additional *in vivo* and *in vitro* experiments to examine the functional mechanisms of the ceRNA network. Third, these results need to be validated in large-scale clinical trials.

## CONCLUSIONS

Taken together, our results suggest that UBE4B expression was increased in HCC and was correlated with a poor prognosis in patients with HCC. UBE4B expression was positively correlated with immune cell infiltration and immunomodulators, chemokines, and their receptors, further suggesting that UBE4B plays a carcinogenic role by modulating immune processes. Additionally, we identified the indispensable role of the UBE4B-hsa-miR-22-3p-FGD5-AS1/LINC00858/SNHG16 regulatory axis in HCC. These findings will aid in understanding the relevant functions of UBE4B and provide new strategies for drug development and exploration of prognosis-related biomarkers.
